# Impacto de un Nuevo Programa de Trabajo Social en el Acceso a la Atención Terciaria

**DOI:** 10.5334/aogh.3886

**Published:** 2022-06-28

**Authors:** Athanasios Burlotos, Paola Alejandra Vargas Díaz, Manuel Alejandro Hernández Pacheco, Lorena Daniela Ponce de León Angel, Miriam Morales Camas, Jesús Sepulveda-Delgado, José Manuel Pérez-Tirado, Santiago Ortiz-Barragan, Anthony T. Fuller, Gustavo Nigenda

**Affiliations:** 1 Duke University Global Health Institute, Durham, North Carolina, USA; 2 Duke University Medical School, Durham, North Carolina, USA; 3 Compañeros en Salud, Jaltenango de la Paz, Chiapas, México; 4 Instituto Nacional de Pediatría, Ciudad de México, México; 5 Hospital Regional de Alta Especialidad Ciudad Salud, Tapachula, Chiapas, México; 6 Universidad Nacional Autónoma de México, Ciudad de México, México

## Abstract

**Antecedentes:**

En el movimiento a favor de la equidad sanitaria mundial, el aumento en investigación y financiación no ha contemplado aun la escasez de evidencias en la aplicación eficaz de las intervenciones en entornos específicos, una necesidad no cubierta es la de facilitar el acceso a la atención especializada dentro del sector de la salud pública en México. Compañeros en Salud ha estado dirigiendo un programa novedoso, llamado Derecho a la Salud (DS), destinado a aumentar el acceso a la atención especializada para poblaciones en situación de pobreza del medio rural en Chiapas, México. El programa DS incorpora trabajo social, seguimiento de pacientes, Referencias, apoyo económico directo y acompañamiento para pacientes.

**Objetivos:**

Este estudio evalúa la efectividad del programa DS. Los primeros resultados analizados incluyen la aceptación de cualquier Referencia y la asistencia a la cita programada. Los resultados secundarios incluyen la aceptación de la primera referencia y la tasa de asistencia a la cita para los pacientes con una referencia aceptada.

**Métodos:**

Utilizando datos del proceso de referenica durante los años 2014 al 2019 de un hospital público de atención terciaria en Chiapas, se empataron 91 pacientes inscritos en el programa DS utilizando una coincidencia de pares óptima 2:1 con una cohorte de control que equilibra las covariables de edad del paciente, sexo, especialidad a la que se remite, nivel del hospital de origen y municipio.

**Hallazgos:**

Los pacientes con DS tuvieron más posibilidades de haber tenido una referencia aceptada (OR 17,42; IC del 95 % 3,68 a 414,16) y de haber asistido a una cita (OR 5,49; IC del 95 % 2,93 a 11,60) en comparación con el grupo de control empatado. Los pacientes inscritos a DS también tuvieron más posibilidades de que se aceptara su primera referencia (OR 2,78; IC del 95 % 1,29 a 6,73). Entre los pacientes con una referencia aceptada, los pacientes pertenecientes a DS tuvieron más probabilidad de haber asistido a una cita (OR 3,86; IC del 95 % 1,90 a 8,57).

**Conclusiones:**

Los resultados demuestran que el modelo DS es exitoso al aumentar el acceso a la atención especializada, tanto en el incremento de referencias aceptadas como en la asistencia a citas.

## Introducción

### Antecedentes

En 2015, las Naciones Unidas presentaron los objetivos de desarrollo sostenible (ODS). El tercer objetivo contempla la salud y el bienestar en todas las edades [1]. Si bien se han logrado avances, ha quedado claro que los métodos utilizados para fomentar mejoras iniciales en la carga mundial de la enfermedad (GBD, por sus siglas en inglés) no serán suficientes para alcanzar el ritmo marcado por los ODS. En particular, los colaboradores de los ODS de la GBD 2017 destacan que será necesario reemplazar las intervenciones terapéuticas que condujeron el progreso inicial por “medidas políticas e inversiones orientadas a la prevención para lograr los objetivos de los ODS” [2]. Para ello será preciso invertir en el reforzamiento de los sistemas de salud existentes. Si bien el aumento en investigación ha permitido documentar la inequidad en materia de salud mundial y la necesidad de actuar, la escasez de investigaciones sobre implementación, especialmente la investigación de implementación en contexto específico, dificulta el avance hacia la equidad en salud mundial [3]. El aumento de la investigación en salud global aún no ha ofrecido pruebas suficientes sobre cómo implementar intervenciones de salud global, para las cuales se ha contado con una financiación cada vez mayor [3].

México, a pesar de ser la 15^a^ economía del mundo clasificada por PIB con rápido crecimiento [4,5], soporta niveles cada vez más altos de desigualdad [6]. Así lo demuestra su coeficiente GINI de 0,45, clasificando a México como un país con alta desigualdad [7]. La pobreza está distribuida geográficamente y afecta de forma desproporcionada a la población indígena, ejemplificada por la superposición entre los estados más pobres y los estados con la mayor proporción de gente de habla indígena [8,9]. Según el PIB per cápita, Chiapas es tanto el estado más pobre como el que cuenta con mas población indigena [8,9]. El índice medio de desarrollo humano municipal en Chiapas es de 0,664, muy inferior a la media nacional de 0,762 [10].

### El sistema sanitario mexicano

La prestación y el financiamiento de servicios sanitarios en México se puede agrupar en tres categorías de pagador y proveedor: seguro social, sector público y sector privado. Los regímenes de seguridad social integrados de pago-prestación están vinculados a la situación laboral. Estos programas abarcan a los trabajadores del sector regulado e incluyen programas como el IMSS (trabajadores regulados del sector privado, 33 % de la población [11(p17))] y el ISSSTE (empleados del gobierno federal, 7 % de la población [11(p17))]. Existen planes de seguro social adicionales para empleados del gobierno estatal, militares y empleados de PEMEX. Cada plan de seguro es una entidad financiera separada y utiliza una red vertical separada de proveedores y hospitales. El sector público se financia a través de los ingresos fiscales generales federales y estatales y administra su propia red vertical de infraestructuras de asistencia sanitaria. El sector público ofrece un paquete de beneficios limitados a quienes no tienen seguro social, esto se analiza con más detalle a continuación. Por último, el sector privado presta servicios de pago por servicio. Si bien solo una minoría de la población con seguro privado depende completamente de este sector para la atención ambulatoria y hospitalaria, el sector privado se utiliza a menudo debido a las barreras de acceso o carencias de disponibilidad de los servicios en el sector público [12].

### El sector público en México

México tiene como objetivo proporcionar cobertura sanitaria universal a través del sector público. Esto comenzó con el establecimiento de la Protección Social del Sistema Sanitario y su rama financiera y operativa “Seguro Popular”, lanzada en 2004 en respuesta a la inequidad financiera dentro del sistema sanitario en México. El Seguro Popular fue el primer impulso hacia la cobertura sanitaria universal en México y proporcionó servicios a una gran parte de la población [13]. El programa tenía 53,5 millones de ciudadanos inscritos en diciembre de 2018, lo que representa el 44,7 % de la población [14,15]. Aunque el Seguro Popular fue cancelado oficialmente en 2018, la asistencia sanitaria universal sigue siendo el objetivo señalado a medida que el sector público se somete a una reforma [16]. (Debido a un retraso en la implementación, los autores no creen que las reformas sanitarias afectaran significativamente a la población del estudio durante el período de su realización.) La naturaleza segmentada del sistema sanitario mexicano conduce a una distribución desigual de la disponibilidad y la calidad de los servicios. Esto es más evidente en pacientes que dependen completamente del sector público, cuya cobertura es limitada en comparación con los planes del seguro social. Por ejemplo, aunque el Seguro Popular financió un total de 56 intervenciones hospitalarias de alto costo, no cubrió el tratamiento de infartos de miocardio en mayores de 65 años o la diálisis crónica [13]. Los recursos sanitarios mexicanos, que son en total inferiores a la media de la OCDE [11(p xxvi)], están especialmente limitados en las zonas rurales. Esto es debido a una infraestructura sanitaria altamente centralizada, con solo un 3,3 % de los hospitales situados en zonas rurales [11(p102)].

En Chiapas, estas desigualdades se agravan para casi el 75 % de la población que depende del sector público para la asistencia sanitaria [17], y son especialmente limitantes para el acceso a la atención especializada. Una métrica útil es el porcentaje de la población registrada en el Ministerio de Salud u otro plan de seguro sanitario, que hasta las reformas recientes era necesario para acceder a la atención especializada. Aunque hasta 2018 la prueba de registro en el Ministerio de Salud era un requisito establecido para acceder a todos los niveles de asistencia sanitaria, en la práctica la falta de registro rara vez impedía que un paciente accediera a los servicios de nivel de atención primaria; sin embargo, el registro en el Ministerio de Salud u otro pagador era un requisito estricto para acceder a la atención secundaria o terciaria. Por lo tanto, el porcentaje de la población sin registrar en ningún servicio sanitario, un indicador del gobierno, es una métrica útil para estimar la población sin acceso a la atención especializada. En 2020, el porcentaje de la población sin registrar en ningún servicio sanitario fue del 26,5% a nivel nacional [15], y del 33,3% en el estado de Chiapas [17]. Para la población rural, el acceso a la atención especializada está aún más restringido por el limitado personal hospitalario, la deficiente infraestructura civil y hospitalaria, las grandes distancias geográficas y la limitada disponibilidad de suministros médicos. Además, la misma falta de economía regulada que impulsa a la población en situación de pobreza de las zonas rurales a depender de los servicios del sector público (ya que están excluidos en gran medida de los empleos asociados a los planes de seguridad social), les predispone a las condiciones económicas que los hacen menos preparados para superar estas barreras.

### Compañeros en Salud y el programa Derecho a la Salud

La falta de disponibilidad de servicios sanitarios para los pacientes en Chiapas llevó a la ONG internacional Partners in Health a colaborar con la Secretaría de Salud local a través de su afiliado, Compañeros en Salud, en las regiones de Frailesca y Sierra Madre de Chiapas en 2011 [18]. Desde entonces, Compañeros en Salud ha podido dotar de personal y apoyo a 11 clínicas sanitarias rurales vacías, aumentando así el acceso a la atención primaria en la región [19]. A pesar de este aumento en el acceso a la atención primaria, todavía existen barreras significativas, a menudo prohibitivas, para los pacientes de la región cuando buscan acceso a la atención especializada. Estos factores incluyen la intersección de la desigualdad económica, los factores geográficos, la limitada infraestructura civil y sanitaria, la discriminación social y cultural y la dificultad para navegar por un sistema sanitario altamente complejo. Además, la población indígena en México, que es prominente en la región de estudio, enfrenta barreras adicionales como la falta de traductores de lenguas indígenas y un mayor aislamiento geográfico [20]. Para ayudar a los pacientes rurales a acceder a la atención especializada, Compañeros en Salud implementó un sistema de apoyo integral y gratuito conocido como el programa Derecho a la Salud (DS). El programa DS contempla las barreras que enfrentan los pacientes del medio rural dentro de la red de atención primaria de Compañeros en Salud al acceder a los servicios de atención secundaria y terciaria en el sector público, a la que todos los ciudadanos mexicanos tienen derecho. Dada la forma en que las barreras de la región reflejan las barreras que enfrentan los pacientes en el sector de salud pública de México en general, las regiones de Frailesca y Sierra Madre de Chiapas son adecuadas para estudiar este nuevo modelo de incremento del acceso a la atención especializada a través del refuerzo y el apoyo al sector público existente.

DS ofrece servicios para ayudar a los pacientes a superar las barreras socioeconómicas a la asistencia sanitaria y contempla directamente las deficiencias de sus prestaciones en el sector público. El programa es innovador y único en el sentido de que contempla de manera integral las barreras a la asistencia sanitaria, financiando los costos médicos y no médicos de los pacientes, coordinando los servicios de trabajo social y proporcionando un apoyo de seguimiento y acompañamiento al paciente. El programa DS utiliza principalmente los beneficios existentes del sector público de los pacientes, pero también ofrece financiación para obtener servicios de asistencia sanitaria cuando la carencias en disponibilidad o cobertura hacen que el servicio no esté disponible en el sector público [21]. Además, el programa DS es único en el sentido de que es un programa integrado de trabajo social y seguimiento del paciente ubicado en el nivel primario de atención en un área rural. Esto contrasta con otros departamentos de trabajo social en México, que se encuentran en hospitales urbanos más grandes. Un modelo económico teórico publicado recientemente de un subconjunto de pacientes del programa DS predice un impacto y una rentabilidad significativos, con un promedio de 14,8 Años de Vida Ajustados por Calidad (AVAC) adicionales para los pacientes incluidos, a un costo por AVAC de 388 dólares americanos [22]. Este prometedor análisis preliminar, la singularidad del modelo del programa DS y la escasez de pruebas en la documentación sanitaria global sobre intervenciones efectivas para aumentar el acceso a la atención especializada mientras se trabaja con los sistemas sanitarios existentes, demuestran la necesidad de un análisis de resultados empíricos del programa DS.

### Objetivos

**Figura 1:**
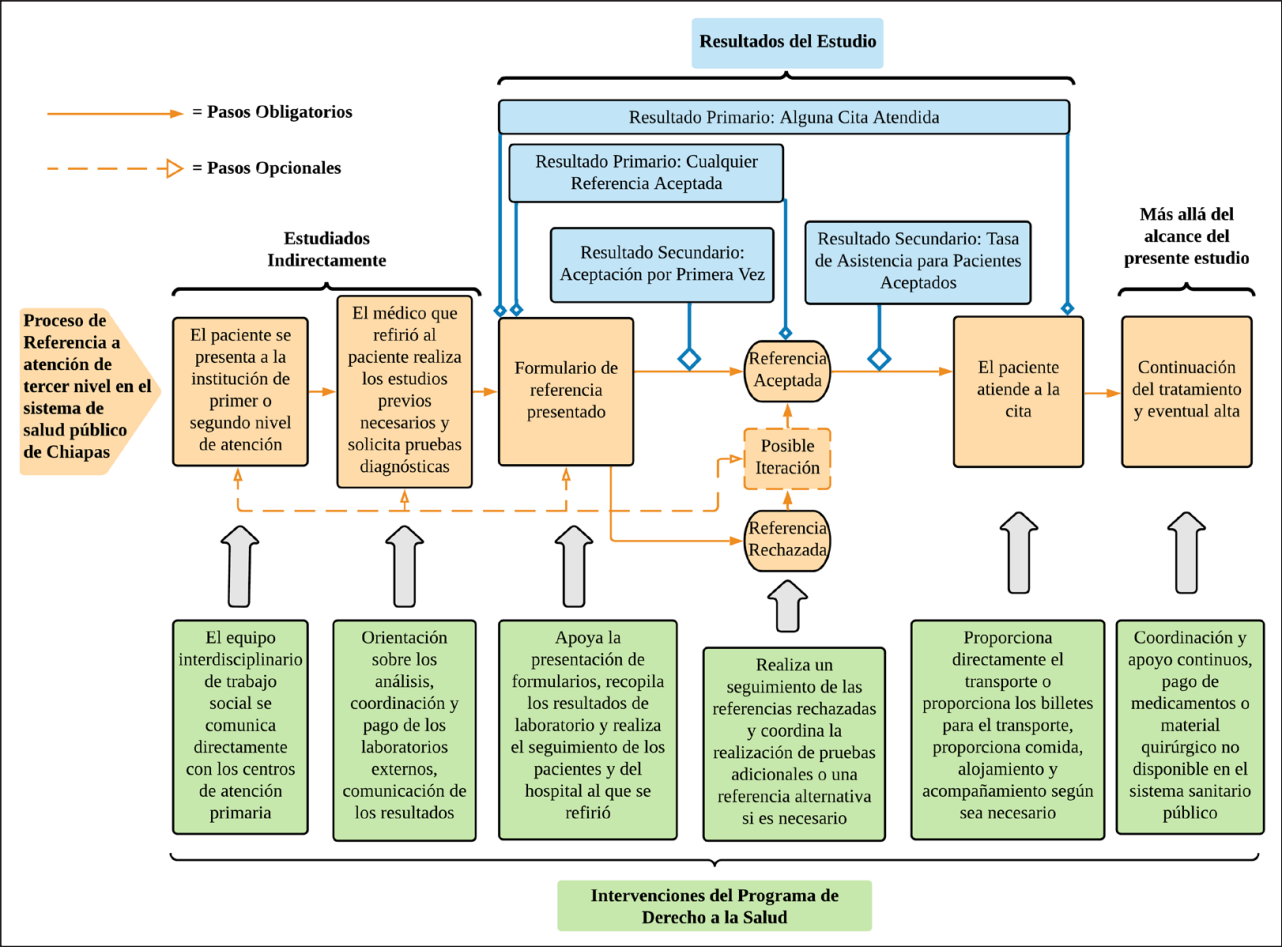
Resumen de las relaciones entre el proceso de referencia, las intervenciones del programa DS y los resultados del estudio.

Con la evaluación de la implementación de un novedoso programa de asistencia social, el estudio busca evaluar la efectividad de este nuevo modelo en el incremento del acceso a la atención especializada en el entorno de la Chiapas rural. La relación entre el proceso de referencia en Chiapas, las intervenciones del programa DS y los resultados del estudio se detallan a continuación en la ***Figura 1***.

## Métodos

### Ética

Se recibió la aprobación de la Junta de Revisión Institucional (IRB, por sus siglas en inglés) de la Universidad de Duke (Número: 2021-0231). Asimismo, la aprobación bioética se obtuvo por medio de la Secretaría de Salud del Estado de Chiapas (firmado por MTRA. Marianna Lazos Salgado). Por último, se concedió la aprobación federal por parte del Comité de Investigación del Hospital Regional de Alta Especialidad “Ciudad Salud” (Número de registro: HRAECS-DGA/JDID/CI/004/2021).

### Entorno

Compañeros en Salud gestiona 11 clínicas de atención primaria en las regiones rurales y montañosas de Frailesca y de Sierra Madre en Chiapas, México. Estas clínicas atienden un área geográfica de 122.469 personas, concentrándose aproximadamente alrededor de la ciudad y el centro municipal de Jaltenango de la Paz, donde Compañeros en Salud tiene su base. En esta región hay un hospital de segundo nivel de atención. No obstante, debido a dificultades con las infraestructuras y la dotación del personal, el hospital es incapaz de proporcionar atención secundaria estándar. No existen centros de atención terciaria en la región.

El Hospital Regional de Alta Especialidad “Ciudad Salud” (en adelante “Hospital Ciudad Salud”) es un centro federal público de atención terciaria situado en Tapachula, la segunda ciudad más grande de Chiapas. El Hospital Ciudad Salud es la única instalación de atención terciaria para adultos del sector público en Chiapas y atiende pacientes de todo el estado y de los estados vecinos. Dada la geografía e infraestructura, un viaje de ida hasta el Hospital Ciudad Salud desde otras comunidades, a las que Compañeros en Salud atiende, dura más de 12 horas en vehículo privado, y entre 1 y 3 días en transporte público.

### Recopilación de datos

**Figura 2:**
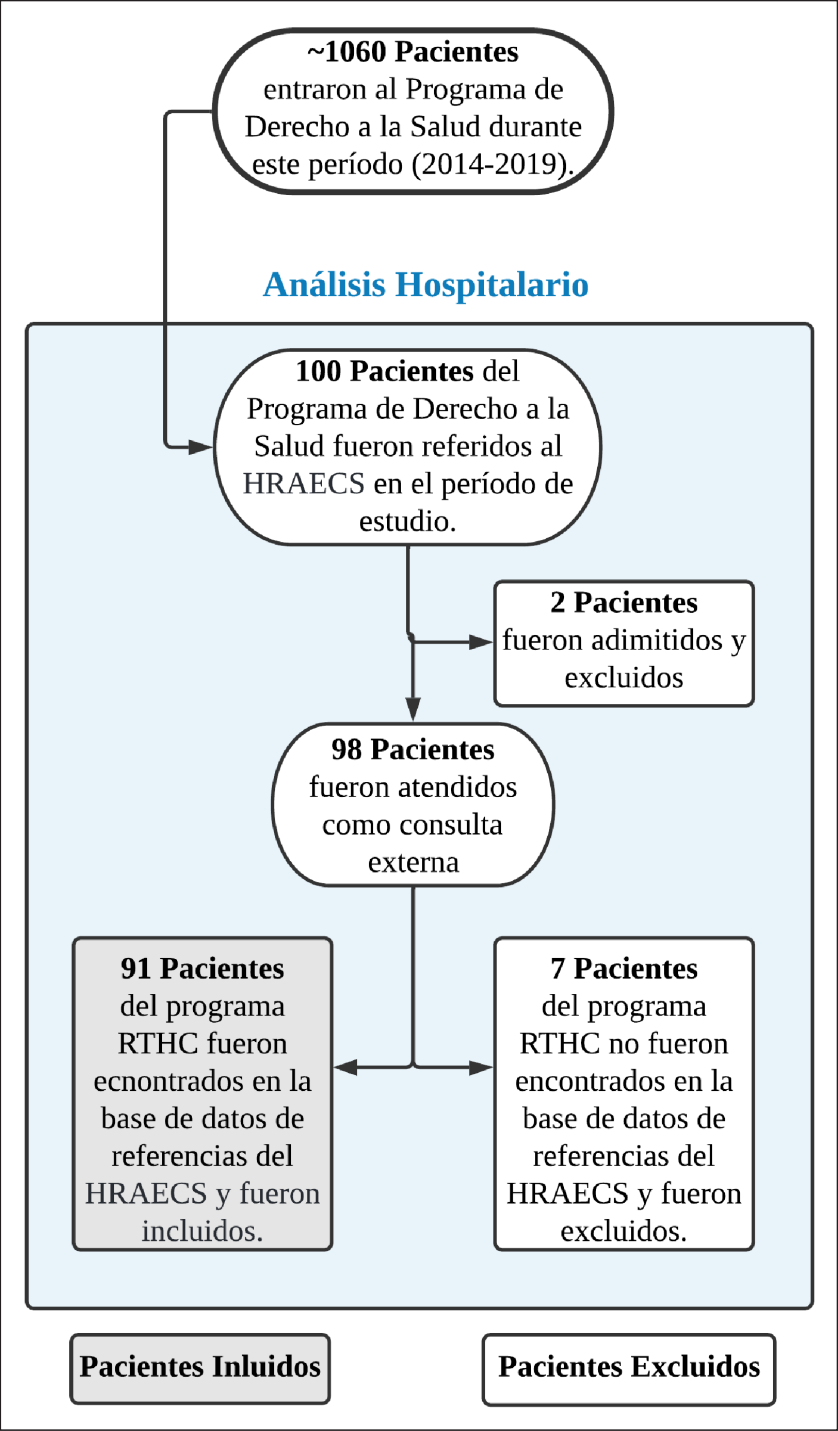
Criterios de inclusión y recuentos de pacientes. HRAECS = Hospital Regional de la Alta Especialidad Ciudad Salud.

El presente estudio fue un análisis retrospectivo para examinar el impacto del programa DS en los resultados del proceso del sistema de referencia en atención terciaria. La recopilación de datos comenzó usando bases de datos internos mantenidos por el programa DS. Todos los pacientes inscritos en el programa DS desde el año 2014 hasta el 2019 y que se identificaron para su referencia al Hospital Ciudad Salud se consideraron aptos para el estudio (100 pacientes). Los historiales se emparejaron con las bases de datos de las referencias del Hospital Ciudad Salud para estos 100 pacientes. Los historiales de siete pacientes no pudieron localizarse en el Hospital Ciudad Salud y se excluyeron. Se sospecha que las referencias de estos siete pacientes no se entregaron al Hospital Ciudad Salud, dado que cualquier referencia que se recibe queda registrada (independientemente de si ha sido aceptada o rechazada). Es probable que estos pacientes se retiraran del programa DS, buscaran atención en otro lugar, o fallecieran antes de que su referencia fuera entregada. Ya que no se asoció exclusión alguna con el resultado, el equipo de investigación consideró que estos números eran aceptables y procedieron con el análisis. De los 93 pacientes restantes, 2 se admitieron en el hospital y recibieron tratamiento hospitalario, y el resto de pacientes se trataron de forma ambulatoria. Debido a las significativas diferencias en los procesos de referencia para pacientes admitidos en comparación con los que recibieron tratamiento ambulatorio, estos dos pacientes también se excluyeron, dejando un grupo de estudio final de 91 pacientes. Los criterios de inclusión y los recuentos están resumidos en la ***Figura 2***.

Las bases de datos del Hospital Ciudad Salud fueron la fuente de todas las variables usadas en el análisis. Primero, las bases de datos de referencias anuales se agruparon y se limpiaron usando Tableau Prep (Versión 2021.1). Las variables extraídas directamente incluyen el nombre del paciente, sexo y edad, así como el resultado de la referencia, la fecha de recepción, la fecha de respuesta y la fecha de la cita si procedía. La especialidad a la que se remitió también se recopiló y agrupó en categorías clínicamente relevantes para facilitar la compatibilidad con el paciente. La ubicación del paciente se obtuvo de un campo de texto libre introducido manualmente y agrupado en distritos oficiales. El nivel del centro de salud referido se obtuvo usando directrices de nomenclatura descritas por el sector público [23], usando información publicada por el respectivo centro y mediante charlas con los médicos locales.

El conjunto de datos se adjuntó con dos fuentes adicionales de información. En primer lugar, se marcaron los pacientes que reunían los criterios de inclusión y estaban inscritos en el programa DS, usando una comparación manual del nombre del paciente, edad y fecha de referencia en los registros internos de Compañeros en Salud. En este punto se agruparon los pacientes. Para los 91 pacientes de DS que reunían los criterios de inclusión y para el grupo coincidente, se recopilaron puntos de datos adicionales desde la base de datos del registro de pacientes del Hospital Ciudad Salud. Cada paciente que asiste a una cita en el Hospital Ciudad Salud se introduce en esta base de datos después de su cita y se le asigna un número de historial de paciente único. Este número solo se asigna a los pacientes después de asistir a su primera cita con el médico. Si la referencia de un paciente se acepta y se asigna una fecha para la cita, pero el paciente no asiste a la cita por cualquier motivo, dicha persona no tendrá un número de historial de paciente. Por lo tanto, la presencia de un número de historial de paciente permitió al equipo de investigación ver qué pacientes asistieron físicamente a sus citas. El conjunto de datos del estudio para pacientes incluidos y emparejados se adjuntó teniendo en cuenta si el paciente tenía un número de historial de paciente y la fecha de registro. Esto se realizó, como se ha citado antes, mediante revisión manual. Cada revisión de datos manual se confirmó con un segundo investigador.

### Análisis de datos

Antes del emparejamiento, el conjunto de datos del paciente que se incluyó, se procesó más a fondo para asignar a cada paciente en la base de datos una identificación única y a los pacientes de grupo con múltiples derivaciones a la misma identificación única. Se excluyeron veintiocho entradas del conjunto de datos del Hospital Ciudad Salud debido a la presencia de valores nulos (0,5 % del total de las entradas, todas de potenciales grupos de control). Acto seguido, se efectuaron cálculos de potencia en R (Versión: 3.6.0) y se seleccionó el emparejamiento de pacientes de 2:1 para mejorar la potencia estadística dentro de los límites de tiempo y recursos del equipo de investigación. El emparejamiento de los pacientes del programa DS que no tienen controles de DS se hizo usando el paquete “Matchit” en R. Los controles se definieron como pacientes derivados al Hospital Ciudad Salud como consultas externas, quienes no estaban afiliados al programa DS. El emparejamiento óptimo se realizó asignando dos controles a cada paciente en el programa DS, compensando las covariables que pudieran afectar al éxito del paciente en el proceso de derivación. Las covariables se seleccionaron basándose en conversaciones con médicos y la disponibilidad de datos, e incluyeron la edad, sexo, especialidad a la que se derivó, el nivel del hospital de derivación y el municipio del paciente. Dado que el Hospital Ciudad Salud es un hospital del sector público, todos los pacientes del estudio no tienen un sistema de seguridad social o un seguro privado y dependen exclusivamente de los beneficios de su sector público. El emparejamiento óptimo de a pares se seleccionó dado un balance levemente mejorado en comparación con el vecino de emparejamiento más cercano. Las características del grupo de control antes del emparejamiento, los controles emparejados y el tratamiento del grupo se comparan abajo en la ***Tabla 1***. El análisis de los resultados se hizo en R usando el paquete “epitools”.

**Tabla 1:** Resumen de los resultados de emparejamiento.


		EMPAREJADOS	
	
COVARIABLES	TODOS LOS CONTROLES APTOS (N = 4470)	CONTROL (N = 182)	TRATAMIENTO (N = 91)

**Especialidad a la que se derivó**			

Oncología	16,2 %	33,5 %	39,6 %

Medicina interna	29,2 %	21,4 %	20,9 %

Otras subespecialidades quirúrgicas	11,8 %	17,0 %	16,5 %

Cirugía general	8,9 %	7,7 %	7,7 %

Neurocirugía	6,0 %	10,4 %	4,4 %

Urología	10.1 %	3,3 %	4,4 %

Ginecología	4,7 %	1,7 %	2,2 %

Neurología	4,6 %	2,8 %	2,2 %

Hematología	0,0 %	0,0 %	1,1 %

Traumatología	6,7 %	2,2 %	1,1 %

**Sexo**			

Mujer	58,7 %	47,8 %	51,7 %

Hombre	41,3 %	52,2 %	48,4 %

**Edad**			

Promedio de edad en años (Rango)	49,9 %	51,9 % (16 – 89)	51,4 % (20 – 86)

**Nivel del hospital de derivación**			

Primario	36,1 %	41,2 %	47,3 %

Secundario	49,7 %	24,2 %	13,2 %

Terciario	1,4 %	1,1 %	1,1 %

Desconocido	12,8 %	33,5 %	38,5 %

**Municipio**			

Ángel Albino Corzo	1,2 %	24,7 %	35,2 %

Siltepec	7,8 %	41,8 %	26,4 %

La Concordia	1,3 %	18,1 %	17,6 %

Montecristo de Guerrero	0,4 %	8,8 %	12,1 %

Capitán Luis Ángel Vidal	0,0 %	0,6 %	1,1 %

Chiapa de Corzo	1,6 %	1,1 %	1,1 %

Chicomuselo	2,8 %	0,0 %	1,1 %

Escuintla	9,1 %	0,6 %	1,1 %

Motozintla	24,9 %	1,7 %	1,1 %

Pijijiapan	33,0 %	2,8 %	1,1 %

Tuxtla Gutiérrez	16,3 %	0,0 %	1,1 %

Villa Corzo	1,5 %	0,0 %	1,1 %


### Resultados

Los resultados primarios analizados fueron sobre si al paciente se le había aceptado alguna derivación (independientemente del número de iteraciones) y sobre si el paciente había asistido a alguna cita. Los resultados secundarios fueron sobre el impacto del programa en la frecuencia de aceptación de la primera derivación y el impacto directo del programa en el índice de asistencia de citas para pacientes cuya derivación fue aceptada. La aprobación de la primera derivación se analizó de manera independiente como un objetivo secundario para averiguar en qué parte del proceso de derivación el programa DS estaba generando un impacto, que permitiera un análisis independiente del impacto de la calidad de la referencia inicial y los efectos del seguimiento, modificación y el reenvío de derivaciones rechazadas. El índice de asistencia para pacientes cuya derivación fue aceptada se analizó de manera independiente para delinear resultados debidos al apoyo en diferentes etapas del proceso de derivación. En concreto, esto separa por un lado los efectos del programa DS sobre la aceptación de derivaciones y, por el otro, los efectos sobre la asistencia presencial a las citas médicas. Por último, se incluyeron recuentos descriptivos para pacientes cuya derivación inicial no fue aprobada, para entender en qué parte del proceso de derivación el programa DS estaba generando un impacto.

## Resultados

La derivación se aceptó en 90 de los 91 pacientes de DS (99 %), comparado con los 149 de 182 pacientes en el grupo de control de emparejamiento (82 %). La probabilidad de que se aceptara alguna derivación fue significativamente más alta para los pacientes dentro del programa DS en comparación con el grupo de control (OR 17,42, 95 % CI de 3,68 a 414,16). Al revisar los registros de los pacientes, 80 de los 91 pacientes de DS habían asistido a una cita durante el periodo del estudio (88 %), en comparación con los 103 de los 182 pacientes del grupo de control de emparejamiento (57 %). Las posibilidades de asistencia a una cita fueron mucho más altas para los pacientes de DS en comparación con el grupo de control (OR 5,49, 95 % CI de 2,93 a 11,60).

La primera derivación se aceptó en 83 de los 91 pacientes en el grupo DS (91 %), comparado con los 143 de los 182 pacientes en el grupo de control de emparejamiento (79 %). Las probabilidades de que una derivación fuera aceptada por primera vez fueron mucho más altas para los pacientes dentro del programa DS en comparación con el grupo de control (OR 2,78, 95 % CI de 1,29 a 6,73). El análisis de subconjunto de los pacientes aceptados obtuvo las diferencias en el índice de asistencia de citas con 80 de los 90 pacientes aceptados por el programa DS que asistieron a una cita (89 %), en comparación con los 100 de 149 pacientes aceptados(67 %) en el grupo de control de emparejamiento (OR 3,86, 95 % CI de 1,90 a 8,57).

Cuando observamos los recuentos de pacientes para saber qué parte del programa DS está generando un impacto, además de los resultados mostrados arriba, cabe destacar que de los 8 pacientes del programa DS cuya derivación había sido rechazada al principio, todos volvieron a solicitarla, y 7 de las 8 derivaciones fueron aceptadas después de las iteraciones (100 % y 88 % respectivamente). En comparación, de los 39 pacientes que habían sido rechazados al principio en el grupo de emparejamiento, solo 7 volvieron enviar la solicitud, y 6 de las 7 derivaciones fueron aprobadas después de la iteración. (18 % y 86 % respectivamente). Los resultados están resumidos abajo en la ***Tabla 2***.

**Tabla 2:** Resumen del estudio de los resultados.


RESULTADO	GRUPO DS (TRATAMIENTO)	GRUPO EMPAREJADO (CONTROL)	RAZÓN DE MOMIOS	CI DEL 95 %

**1.1** : Cualquier derivación aceptada	99 % (90/91)	82 % (149/182)	17,42	3,68 – 414,16

**1.2** : Cualquier cita atendida	88 % (80/91)	57 % (103/182)	5,49	2,93 – 11,60

**2.1** : Derivación aceptada por primera vez	91 % (83/91)	79 % (143/182)	2,78	1,29 – 6,73

**2.2** : Índice de asistencia para pacientes aceptados	89 % (80/90)	67 % (100/149)	3,86	1,90 – 8,57


## Discusión

Este estudio es el primer análisis de resultados del programa DS, que representa un novedoso modelo de asistencia social, el cual podría adaptarse para mejorar el acceso a la atención especializada en cualquier lugar rural de México, en países de ingreso medio y bajo(LMICs, por sus siglas en inglés), o poblaciones vulnerables en países de ingreso alto(HICs, por sus siglas en inglés). Los resultados del estudio son positivos. Esto refleja tanto la magnitud de las barreras a las que se enfrentan los pacientes cuando buscan atención especializada en la región como el éxito del programa DS a la hora de ayudar a los pacientes a superar estas barreras. Es importante interpretar estos resultados dentro del contexto del proceso de derivación a la atención terciaria en Chiapas, y los diversos puntos en los que los pacientes se enfrentan a barreras de acceso.

Como se ilustra en el diagrama, los resultados resaltan el impacto del programa en los diferentes pasos del proceso de derivación. El programa DS facilita el inicio del proceso de derivación, orienta a los médicos que presentan los formularios de derivación, y coordina y proporciona apoyo económico para los estudios de diagnóstico. Aunque no se evaluó individualmente, el colectivo de estas intervenciones puede verse en el resultado primario de haber tenido alguna derivación aceptada, ya que los pacientes tenían mayores probabilidades de tener una derivación aceptada si estaban inscritos en el programa DS. Del mismo modo, el resultado secundario de que se acepte la derivación por primera vez también demuestra una probabilidad significativamente mayor de aceptación para los pacientes del grupo de intervención. Ambos resultados hablan de calidad mejorada de las derivaciones que se presentan. Los mecanismos más probables detrás de estos resultados mejorados son una sinergia de las intervenciones que se acaban de discutir, facilitadas por un equipo de derivación interdisciplinario dedicado a través del programa DS. El apoyo económico y la coordinación con los laboratorios privados garantizan que los resultados de laboratorio y los estudios de imagen adecuados acompañen al formulario de derivación. Por último, el conocimiento institucional de los hospitales de la región a través del equipo de trabajo social facilita la derivación al hospital adecuado con la documentación requerida.

El siguiente resultado primario, la asistencia a cualquier cita, demuestra que los efectos observados en la tasa de aceptación de derivaciones condujeron a un aumento significativo de la asistencia a las citas para el programa DS, en comparación con el grupo de control emparejado. De todos los resultados estudiados, este es el que demuestra de manera más directa que el programa DS está generando el impacto clínico previsto: aumentar el acceso de los pacientes a la atención especializada.

Por último, la tasa de asistencia a las citas para el subconjunto de pacientes a los que se les aceptó la derivación arroja una importante distinción en los resultados. Se demostró un aumento significativo de las probabilidades de que los pacientes del DS con una derivación aceptada asistan a una cita, en comparación con los pacientes del grupo de control emparejado con una derivación aceptada, y así, en este resultado se muestra que el programa DS es eficaz de manera independiente para aumentar las probabilidades de que un paciente asista a su cita. En otras palabras, este resultado demuestra que el impacto del aumento de la asistencia a las citas no solo se debe a un aumento de la aceptación de las derivaciones, sino también a un impacto directo en el aumento del índice de asistencia a las citas de los pacientes. Esto se debe probablemente a la ayuda económica y apoyo a la orientación del paciente que ofrece el programa DS para ayudar a los pacientes a superar las barreras señaladas con anterioridad en la introducción. Además, el acompañamiento de los pacientes a lo largo del proceso pudo haber contribuido a aumentar la tasa de asistencia a las citas.

Para aclarar aún más el impacto del programa en los pacientes rechazados al principio, se destacan otros recuentos descriptivos de pacientes. Sobre todo, hubo una diferencia entre el grupo de tratamiento y el de control en el número de pacientes rechazados que volvieron a solicitar una derivación. Mientras que el 100 % de las derivaciones rechazadas en el grupo del DS se volvieron a solicitar posteriormente, solo el 18 % de los controles emparejados rechazados se volvieron a solicitar, lo que sugiere que una parte del beneficio observado del programa DS proviene de hacer un seguimiento exitoso de las derivaciones rechazadas al principio, de completar los cambios solicitados y de volver a solicitarlas con éxito. Dada la complejidad logística del proceso de derivación, no es de extrañar que un equipo de derivación específico, como el utilizado en el programa DS, mejore el seguimiento, la corrección y el reenvío de los pacientes rechazados. En conclusión, los resultados hablan de la magnitud de las barreras a las que se enfrentan los pacientes de la región, como muestra el hecho de que solo el 57 % de los pacientes derivados en el grupo de control acudieron alguna vez a una cita y el éxito del programa DS a la hora de ayudar a los pacientes a superar estas barreras. Este éxito se demuestra en todos los resultados analizados.

En este estudio se examina un programa integral de apoyo a la derivación, para el cual no hay ningún equivalente documentado en la literatura. No existe un programa comparable en ningún otro lugar de México. Incluso cuando se busca una comparación a nivel internacional, los programas comparables suelen abordar un determinante social de la salud específico no satisfecho. En cambio, el programa del DS es exhaustivo y aborda muchos obstáculos a la atención, que surgen de los determinantes sociales a través del trabajo social, la orientación del paciente, el apoyo financiero y el acompañamiento. Un ejemplo de programa existente son las furgonetas de transporte de Medicaid, en Estados Unidos. En Carolina del Norte, Project Access trabaja para hacer frente a las disparidades en el acceso a la atención especializada; sin embargo, Project Access financia o coordina de manera directa las donaciones para la atención especializada de pacientes sin seguro [24]. Mientras que Project Access atiende a los pacientes que no reúnen los requisitos para tener un seguro gubernamental ni ganan lo suficiente para adquirir un seguro privado, el programa DS trabaja para facilitar el acceso al sector público y cubrir las carencias en los servicios cuando es necesario. Aunque el programa DS es único, los resultados son coherentes con trabajos anteriores que han demostrado el éxito de abordar barreras individuales específicas para el acceso. Por ejemplo, se demostró que cuando se aplicaron los servicios de transporte de Medicaid, aumentó el acceso y disminuyeron los gastos generales, debido a la reducción del uso de los servicios de emergencia y las hospitalizaciones [25]. Aunque en este estudio no se incluyó el ahorro de gastos del sistema de salud, es importante destacar el precedente de que aumentar el acceso puede reducir los gastos generales de la atención sanitaria. Otro ejemplo contundente de los efectos del aumento del acceso se muestra en un estudio reciente sobre pacientes de traumatología en Estados Unidos. Mientras que las disparidades raciales en los servicios de traumatología que hay entre pacientes negros y blancos han sido documentadas exhaustivamente a través de una revisión sistémica y del uso de meta-análisis [26], en este estudio a gran escala se descubrió que entre los pacientes con cobertura sanitaria militar de TRICARE, se eliminaron las disparidades raciales, lo que sugiere que un sistema de atención sanitaria de acceso igualitario podría ayudar a remediar las disparidades sanitarias existentes en los servicios de traumatología [27].

Una de las limitaciones de este estudio es que la covariable de localización equilibrada en el grupo de control emparejado solo pudo emparejarse a nivel de municipios, que es un nivel geográfico más o menos equivalente a los condados en Estados Unidos. En la región estudiada, los municipios se centran en una ciudad que sirve de centro municipal. Mientras que los pacientes de DS provienen de pequeñas comunidades rurales en las montañas, el grupo de control está sesgado hacia ciudades más grandes y centros municipales, debido a la distribución de la población que favorece a esas áreas urbanas. Dado el gran aumento relativo del desarrollo y la proximidad a las infraestructuras y los servicios de salud, en los centros municipales, es probable que el grupo de control no se enfrente a la misma magnitud de barreras que el grupo de tratamiento. La consecuencia de esta limitación es que probablemente se subestime el efecto del tratamiento del DS. El estudio actual también se limitó a un solo hospital de atención terciaria, y se necesitan investigaciones futuras para demostrar hasta qué punto los resultados son transferibles a otros hospitales.

Además, una gran parte de los recursos del DS, tanto en personal como en gastos del programa, se dedica a apoyar a los pacientes a lo largo de su tratamiento. Esto incluye el apoyo constante del trabajo social, la gestión de casos y la cobertura directa de medicamentos y suministros quirúrgicos que no están disponibles a través del sector público pero son necesarios para el tratamiento constante de los pacientes. El impacto de este apoyo constante no pudo medirse en el ámbito de este estudio y debería ser una prioridad para futuras investigaciones. Las investigaciones futuras también deberían aclarar los efectos de las diferentes modalidades de apoyo ofrecidas por el programa DS. Al comparar los resultados de la ***Tabla 2*** con las intervenciones del programa en la ***Figura 1***, este estudio sugiere que los impactos positivos en los resultados se deben tanto al apoyo a la orientación del paciente (que es el principal modo de apoyo antes de que se acepte la derivación) como al trabajo social y al apoyo financiero (que son los principales modos de apoyo después de que se acepte la derivación). Sin embargo, los autores y el equipo que gestiona el programa DS creen que una gran parte del éxito del programa se debe a la sinergia del apoyo a la orientación del paciente, el trabajo social y el apoyo financiero. Este enfoque integral, basado en la evaluación de las necesidades individuales de los pacientes, impulsa el éxito del programa. Por el contrario, si las intervenciones individuales del programa se administraran de forma aislada, su impacto se vería obstaculizado por las necesidades no satisfechas en otras áreas. Las investigaciones futuras también deberían explorar los efectos de la edad, el grupo de diagnóstico y otras variables en el impacto del programa DS.

Otra sugerencia para futuras investigaciones es la eficacia de utilizar los servicios de telemedicina para aumentar el acceso a la atención especializada en la región. Una reciente revisión mundial destaca los prometedores resultados del uso de la telemedicina para aumentar el acceso a la atención especializada; sin embargo, el único país latinoamericano incluido en la revisión fue Brasil [28]. En conjunto, esto sugiere la necesidad de pilotar y evaluar el uso de la telemedicina para aumentar el acceso a la atención especializada en América Latina, lo cual podría incorporarse al programa DS en el futuro. Por último, las investigaciones futuras deberían evaluar el uso de un programa de derivación similar en diferentes contextos, que incluya a las personas en situación de pobreza de las zonas urbanas, otros países de ingresos medios y bajos, y países de ingresos altos, ya que las barreras a la atención especializada no se limitan al entorno del estudio. Por ejemplo, se informa de la dificultad de acceso a la atención especializada entre los pacientes que se atienden en los centros de salud comunitarios de todo Estados Unidos de América, en especial entre los pacientes sin seguro [29]. La investigación debe centrarse en la evaluación del impacto de la inversión en servicios que aumentan el acceso a la atención especializada, ya que el paradigma actual hace recaer la carga de los gastos, la logística y la gestión de unos sistemas de salud cada vez más complejos, en los pacientes y sus redes de apoyo.

## Conclusión

Un pequeño pero creciente conjunto de pruebas sugiere que el programa DS representa un modelo eficaz y específico para aumentar el acceso a la atención especializada. Dada la singularidad del programa y la eficacia demostrada, los resultados de este estudio abogan por que se siga financiando, ampliando e investigando. Es posible que el programa se adapte para satisfacer con éxito las necesidades de los pacientes en otros lugares de México, en otros países de ingresos medios y bajos, o de poblaciones vulnerables en países de ingresos altos.

## Información de financiación

Duke Global Health Institute Medical Student Research Grant, Wagner Foundation administrado mediante Partners in Health.

## Contribuciones de autor

Todos los autores tuvieron acceso a los datos y a un papel en la redacción del manuscrito.
